# Indigenous food security revival strategies at the village level: The gender factor implications

**DOI:** 10.4102/jamba.v8i2.175

**Published:** 2016-01-13

**Authors:** Wilfred Lunga, Charles Musarurwa

**Affiliations:** 1 African Centre for Disaster Studies, North West University, Potchefstroom Campus, South Africa; 2 Department of Languages and Social Sciences Education, University of Botswana, Botswana

## Abstract

This article is based on an evaluation concerning the practice of the Zunde raMambo concept (commonly referred to as Zunde) in four of Zimbabwe’s 52 districts; (Mangwe, Lupane, Guruve and Hwedza). *Zunde* is a social security system providing protection against food shortages to vulnerable families and is coordinated by chiefs. The *Zunde* concept identifies with Ndebele and Shona rural communities in Zimbabwe. Thus, this evaluation sought to determine the relevance and fulfilment of the *Zunde* project objectives, namely: efficiency, effectiveness, impact and sustainability. The revived *Zunde* practice extends a long way in reducing food insecurity in vulnerable communities. Although the concept may be as old as the Zimbabwean culture, it had been abandoned as communities became urbanised. The Chief’s Council of Zimbabwe, in collaboration with the Nutrition Unit of the Ministry of Health and Child Welfare have rekindled it. However, to revive this indigenous knowledge practice, there is need to assess the nature of existing social and economic structures, leadership, gender roles and the availability of resources such as land, inputs and implements. This article, which is based on both qualitative and quantitative data, collected between September 2013 and March 2014, goes on to reflect on policy issues surrounding disaster risk reduction (DRR) and survival strategies used by vulnerable communities in rural areas of Zimbabwe. It recommends that the gender factor approach offers the best means possible to understand peoples’ needs and challenges as well as how these can be satisfied and resolved respectively.

## Introduction

*Zunde raMambo* draws parallels with European feudal systems where a similar arrangement was used not for food security purposes but for farmers to pay feudal rent to knights or barons who were the land owners. From an African perspective chieftainships (*huMambo*) have their roots in feudalism, although in Africa, unlike in England, feudal traditions and institutions have not been well blended to co-exist with capitalists institutions. However, in Zimbabwe, chiefs have managed to retain their traditional power and hence managed to convince the populace that feudal traditions are part of culture which defines the identity of the population. This has led to the survival of the *Zunde raMambo* concept from one generation to another.

In Zimbabwe many rural communities are vulnerable to drought and, hence, experience hunger and malnutrition from time to time (Chigodora 1997; Gasana *et al*. 2011; Gumbo 2006; Gwimbi 2009). Efforts to reduce this challenge, by development organisations including government, have not yielded much success. The proportion of people suffering from malnutrition, a proxy for food insecurity, has increased (Devereux & Maxwell 2001a; International Food Policy Research Institute [IFPRI] 2000, 2002). This scenario raises many questions about what went wrong with the agricultural revolution technologies such as the use of fertiliser, improved seed, irrigation schemes from the government and donor investments in agricultural research including extension programmes. In Zimbabwe climatic modelling studies show that the annual mean temperature has increased by 0.4 °C whilst rainfall has declined by 5% since 1900, hence the increased frequency of droughts (Eriksen *et al*. 2007; Intergovernmental Panel on Climate Change [IPCC] 2007; Madamombe 2004; Moyo 2011). This in turn has motivated the Council of Chiefs to resuscitate a traditional social welfare practice known as *Zunde raMambo* in Shona (*Isiphala* senkosi in Ndebele) as a way of ensuring food security in rural communities. Besides family owned food production units, the chief sets aside a communal plot where all families work at least once a week. The produce from the communal plot is held by the chief in trust for the community and is intended to be used during disaster periods or special occasions. During such periods priority is given to disadvantaged families, individuals and groups. However, in Zimbabwe, colonialism introduced a cash economy that weakened traditional social support systems (Chigora, Dzinavatonga & Mutenheri 2007; Risiro *et al*. 2012; Tsiko 2011). This was coupled with rural to urban migration of men in the working age group in search of remunerated employment, leaving the dependent sections of the community such as women, children and the aged isolated in rural areas. Food production plummeted as a result of these circumstances and, hence, traditional chiefs rekindled the *Zunde* concept to meet social security needs of vulnerable families. Thus, *Zunde* has become a major community institution providing food security (Stathers *et al*. 2000).

In short, the *Zunde* concept is a traditional practice that identifies with many rural communities within the Ndebele and Shona speaking people (Risiro *et al*. 2012). It must be noted that *Zunde* is only one of the many social systems that exist within the Zimbabwean community. Whilst other social security systems, such as burial societies, offer different types of social insurance, the *Zunde* scheme, according to Stathers *et al*. (2000), provides rudimentary protection against food shortages particularly to vulnerable families within a community. This social security scheme attempts to compensate for the inaccessibility and inadequacy associated with other formal schemes through collective responsibility and the historically rooted extended family system. Thus, the Ministry of Health and Child Welfare, through the Nutrition Unit, has supported the introduction of the *Zunde* in rural communities including the districts of Hwedza, Guruve, Mangwe and Lupane. This is meant to cushion women, children and the elderly, during periods of food shortage, as they are the most vulnerable groups. Nutrition in turn has a bearing on people’s health.

### Factors associated with food insecurity in Zimbabwe

Food security is defined as the success of livelihoods to guarantee access to sufficient food at the household level (Food and Agriculture Organization 2010; Moyo 2010, 2011). The emphasis is on food availability at all times and an active healthy life. Thus, food security is related to poverty reduction and, hence, any factor which reduces the capability of people to access adequate food has an effect on poverty and, broadly, on development (Enete *et al*. 2011; FAO 2008). However, Devereux and Maxwell (2001b:2) warn that food insecurity is not merely a subset of poverty but that the precondition for tackling poverty lies in improving the production, marketing and consumption of food at both national and household levels. Sadly food insecurity and poverty are a permanent feature in Zimbabwe, exacerbated by persistent threats of drought (Moyo 2011; Zimbabwe Government & United Nations Development Programme 2011). Zimbabwe climatic modelling studies show that the annual mean temperature has increased by 0.4 °C whilst rainfall has declined by 5% since 1900, hence, the increased frequency of droughts (IPCC 2007; Madamombe 2004; Moyo 2011). Droughts become an issue because agriculture in Zimbabwe is largely labour intensive and rain fed. Current and future changes in climate, especially rainfall and temperature, are likely to render a large section of the population vulnerable to food insecurity (World Bank 2008).

Besides drought, the AIDS epidemic is another important factor as it reduces agricultural productivity caused by its effects on the productive age group (Clover & Eriksen 2009; Mazzeo 2011). This challenge is particularly more pronounced in Zimbawe’s rural communities which depend on family labour for agricultural production (Chigodora 1997; Chitiga & Chigora 2010; Gasana *et al*. 2011; Mazzeo 2011). Low agricultural productivity leads to food crises and, consequently, to food insecurity. Another challenge is that indigenous knowledge, which could have helped communities in dealing with food security issues, has been ignored and maligned as primitive and simple (Berkes 2009; Chigora *et al*. 2007). Risiro *et al*. (2012) support this perspective by pointing out that most people have a tendency to absorb one form of knowledge, especially the western one, at the expense of the local or indigenous knowledge and this has been detrimental to the wellbeing of the populace.

However, there is no single answer to the multifaceted nature of food security. To understand the degree of vulnerability to food insecurity one must understand the relationships, amongst many factors, such as livelihood strategies, resource endowments, social dynamics, hazards and coping strategies (Clover 2006, Preventionweb, 2012). These influence the availability, access and utilisation of food within a social, political, economic and environmental context at national and household level.

### The role of Chiefs in ensuring food security

For one to appreciate the role of chiefs in food security, one must understand both the traditional and constitutional means by which chiefs derive their power. Visser, Steytler and Machingauta (2010) posit that the function of chiefs in Zimbabwe can be classified into five broad categories, namely:

- constitutional and legislative
- judicial (including the maintenance of law and order)
- ceremonial
- religious
- developmental.

These functions are, broadly, in line with practices in many other parts of Africa. Traditionally chiefs look after the spiritual and material welfare of people under their jurisdiction (Visser *et al*. 2010). Chiefs preside over traditional courts with the assistance of advisors selected from within the community. They also perform a wide-range of ceremonial rituals, including ensuring the observation of traditions for the protection of their own people, the safety of the sacred relics at their disposal, and carrying out rites on behalf of the community (Mushongah 2009; Visser *et al*. 2010). The role of traditional chiefs is, therefore, to protect the livelihood of the people and their environment. Hence, indigenous people continue to exercise some level of self-governance and autonomy (Manyena 2006; Maphosa 1994; Mushongah 2012).

The *Traditional Leaders Act* (Chapter 29:17 of 1998) set out guidelines on how chiefs and their deputies are appointed, and how they ought to relate to other branches of the government (Manyena 2006). Part II, sections 3 to 7 of the same Act, also outlines how chiefs are to be appointed, their duties, and the disciplinary procedures as they apply to chiefs. Thus, chieftainships are recognised by both traditional and secular institutions, hence, chiefs wield much power, making it easy for the *Zunde* concept to be acceptable.

### Gender factor and food security

‘Gender’ refers to socially-constructed roles, behaviours, activities and attributes that a society considers appropriate for a person based on his or her sex (Enarson 2006; Hassan & Nhemachena 2008). Thus, gender implications on natural disasters such as droughts are critical to effective disaster risk reduction (DRR) practices that enable communities to be disaster resilient. Enarson (2006) highlighted that although men, women, girls and boys have different needs and vulnerabilities when disasters strike, it is women and children who are mostly negatively affected. This necessitates appropriate coping strategies in prevention, relief, recovery and reconstruction processes.

Usually the important roles that women take in relief, recovery and reconstruction are often not recognised. Although decisions are made by men including the chief, it is the women who perform most of the cultivation, harvesting and preservation in the *Zunde* fields. It should be noted that Zimbabwe operates a dual economy with two distinct systems; one rural and one urban (Chikoto 2004; GoZ & UNDP 2011; Maphosa 1994). Most men migrate to urban areas seeking industrial jobs and women are left in rural areas to fend for children and themselves (Enarson 2006; Enete *et al*. 2011; Hunter-Gault 2006; Kinsey, Burger & Gunning 1998). Thus, food insecurity affects rural inhabitants more, who incidentally are women and children, and in extreme cases widows and orphans. In short, women play a critical role in agricultural and pastoral livelihoods, often bearing significant responsibility for managing critical productive resources such as land, water, livestock, biodiversity, fodder, fuel, and food. In addition they also contribute work and energy towards income generation and carry out a disproportional amount of daily labour compared to men in a household. Their contribution is also disproportional in community spheres such as cooking, cleaning, child care, care of older or sick family members, providing work for collective projects and during weddings, funerals and other cultural ceremonies.

Empowering women to decision making may increase their coping range, as distinct from temporary and historically familiar measures to cope with a transient threat, and yet the decision making in chieftainships has remained mostly patriarchal (Chitiga & Chigora 2010; Gumbo 2006). The inclusion of women can be a positive adjustment to Zimbabwean society’s response to the perceived vulnerability of food insecurity. After all the success of Zimbabwe’s agriculture system over the years is attributed to women’s roles in achieving food production, income and livelihood security objectives in the face of extreme weather conditions such as droughts and floods.

## Methodology

**FIGURE 1:**
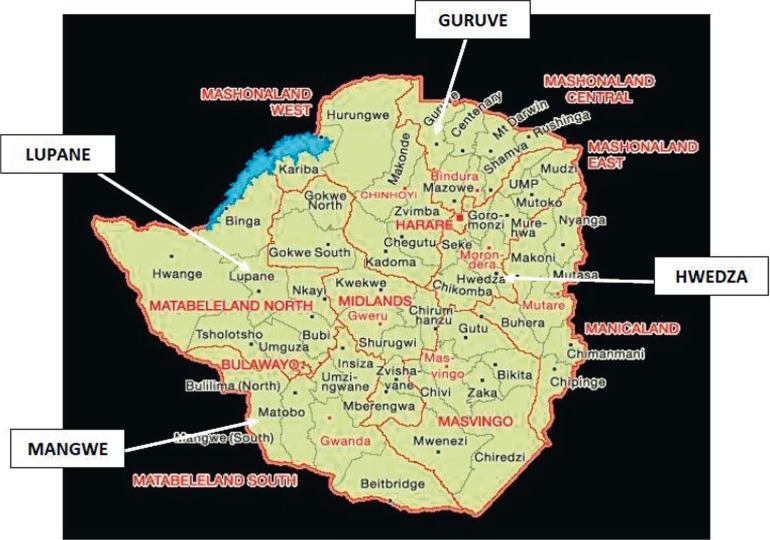
Map of Zimbabwe showing the four districts of study.

The study was conducted in Guruve in Mashonaland Central, Hwedza in Mashonaland East, Lupane in Matabeleland North and Mangwe in Matabeleland South, shown in Figure 1.

The four districts of Guruve, Hwedza, Mangwe and Lupane were purposively selected for the study as they were reviving the *Zunde* practice; they still have a rich cultural heritage, use appropriate indigenous knowledge (IK), and are keen to adopt mitigation measures aimed toward food security. Also, for logistical reasons they were the most convenient areas to conduct research. These areas rely entirely on subsistence farming, which is dependent on natural rainfall. Reliance on subsistence farming additionally means that the inhabitants are seasonally employed or under-employed (Government of Zimbabwe 2011a; United Nations Development Programme 2010). Mashonaland province is home to Shona speaking people (*Karanga, Zezuru and Korekore*) whilst Matabeleland is home to the *Ndebele, Kalanga* and *Tonga* speaking people (Central Statistical Office [CSO] 2012; GoZ & UNDP 2011). Thus, the study’s sample spanned across the country’s population in an effort to establish whether the *Zunde* practice was relevant to all Zimbabweans. To this end the evaluative study used data from primary sources collected through structured questionnaires, in depth interviews, observation; transect walks and focus group discussions. This evaluation assessed the extent to which the *Zunde* project had been received in the chosen study areas after its revival through the recommendations of the Chiefs’ council. The main focus was on available social and economic structures, access to and control of resources, quality of leadership and the relationship which existed between local leadership and government structures.

A blended research design of both qualitative and quantitative approaches was adopted. Focus group discussions were held with an average of 23 people per ward that included the ward councillors, the village headmen, 12 females and 9 male elders from each village, and an official from the district department. Questionnaire based interviews were conducted with 8 traditional leaders and these were selected using purposive sampling (Bryman 2012; Creswell 1998). Questions for traditional leaders centred on reasons for resuscitating the *Zunde,* its relevance and implications for DRR. The respondents in households included men and women depending on their availability at home at the time of conducting interviews. Nevertheless, women were the main respondents on issues pertaining to agricultural practices.

Participatory techniques were used to learn through observation and hands-on experience of the practices and procedures involved in traditional agricultural practices. The aim here was to establish a practical dimension and also to make findings context specific and reflective of reality. The four districts of Guruve, Hwedza, Mangwe and Lupane were purposively selected for the study as they were reviving the *Zunde* practice, cultural heritage, use of IK, and engaged in recent mitigation actions and the nature of collective action or community initiative related to disaster issues. Also, for logistic reasons they were the most convenient areas to conduct the research.

The unit of study was the household then scaled up to the community level to allow an analysis of community participatory approaches and mechanisms. The household as a unit of study was chosen because most decisions about household production, investment and consumption are made at this level in most agrarian societies, particularly under long-lasting drought conditions (Ziervogel *et al*. 2006).

## Findings and discussion

One of the objectives of this evaluative research was to assess the extent to which stakeholders see the relevance of the tradition and how far this agricultural practice makes households food secure. Discussions with traditional chiefs who are the custodians of the practice and keep the production records, indicate that there has been an increase in food production ranging from 0.4 to 4.2 tonnes per hectare across all the project sites. Ironically, this increase in food production is despite the fact that much of the food production in Zimbabwe’s rural areas is cultivated through subsistence labour provided by women and children, whilst most men have fulltime employment in urban areas. This gender factor dichotomy was discussed earlier in the introduction. The relevance of the practice, according to most women respondents in all the districts, was that it provided pooled tillage assets, use of local resources, pooled labour and use of appropriate technology that is more affordable to vulnerable communities. A group of men and women farmers in the Guruve district stated that the Zunde raMambo has contributed to high yields, but this has not in all cases translated into poverty reduction. This is because although there were high yields, they have not provided surplus for selling and trading.

The *Zunde raMambo* practice has also been used as a field school within the community, to local farmers, to reduce the distance they travel to acquire knowledge, thereby contributing to project sustainability. It is also evident that planning and implementation is undertaken using local structures, processes and procedures, under the chief’s guidance, thereby ensuring high community participation and organisation, thus, feeding into programme efficiency. The *Zunde* social security concept leads people to believe in what village elders, headmen and chiefs regard as a tradition and hence people buy into the programme creating a sense of ownership by the community. In the long term this will have a positive impact by increasing food security in a sustainable way.

Findings also reveal that maize is the most popular crop in all the *Zunde* project sites, especially in Guruve and Hwedza. Mangwe and Lupane preferred small grains as these areas are prone to drought. The most important asset in the *Zunde* is the land and seeds which are obtained from the previous harvests and preserved using indigenous knowledge practices such as smoking them with soot and by hanging them in the traditional kitchen roof. Participants also highlighted the important role played by the chiefs in the *Zunde* project by spearheading the use of indigenous technology. Such technology includes conservation farming, water harvesting (to supplement rainfall), and the use of vegetative matter (*murakwane*) and crop waste (*mashanga*) as fertiliser.

Through interviews the chiefs identified that produce from the *Zunde* fields was used to supplement the feeding of infants, the disabled, and the old members of the villages and was also used to support the bereaved. The programme is also being used as a form of insurance or food bank as, occasionally, villagers who had run out of food borrow grain from the *Zunde* granary, to be replaced after the next harvest. Thus, communities can work as partners with joint ownership. The project has also facilitated understanding and effective communication amongst community members.

Traditional chiefs and village elders observed that the concepts of *Zunde* in Shona or *Isiphala* in Ndebele constitute an informal, in-built social, economic and political rallying mechanism. It allows the traditional chief to have control over people under his jurisdiction and share his views amongst the community. Whilst the major aim of the concept is to ensure adequate food reserves that could be used in times of food shortage for a particular community, the chief also used its rallying mechanism over his area of jurisdiction, thus, securing their safety.

Elders with ages ranging from 67 to 81 reiterated that food security for households is guaranteed at all times as a result of the *Zunde*. One elderly women of age 66 had this to say during in depth interviews:

Time shall come when these chiefs are no more, but the knowledge we have gained will remain. They made us taste what is good so this is motivation enough for us to keep striving to maintain the standard and keep practicing the Zunde.

In Mangwe and Guruve, it was observed that it was not only chiefs who practised the *Zunde* but other individual homesteads, especially polygamous homes, had miniature replicas known as *Zunde raBaba* or *Isiphala sabobaba*. Women in the polygamous family are allocated pieces of land they would work with their children to grow crops. Rosters are put in place with days when the whole family works in the husband’s family fields and on the remaining days women and their children work in their own fields. Granaries are separated such that the husband’s granary is only used during times of distress or when women finish their own resources.

Indeed the *Zunde* programme is one practical example of how indigenous knowledge influences the life of traditional communities. Indigenous knowledge also plays an important role in the socioeconomic wellbeing of communities and the management of resources, including the environment, hence the very survival of such indigenous communities. The research findings demonstrated that indigenous knowledge provides the basis for grassroots decision making, much of which takes place at the community level through village elders and traditional leaders including indigenous organisations. Women, although they are not consulted, bring in their labour and knowledge on what crops to grow and, thus, play an important role in implementation. The study also noted that indigenous knowledge not only empowers local communities, but contributes to food self-sufficiency.

This study supports earlier ones regarding the positive impact on the adoption and adaptation of the *Zunde* concept by farmers. Mushongah (2009) point out that in *Zunde* agricultural activities indigenous food production contributes significantly to food security. This is the result of cheap labour provided by women and children with regards to tillage and weeding. Indigenous knowledge, in its various manifestations, also gives cultural pride and motivation to solve local problems with local ingenuity and resources. Thus, it is a crucial aspect of sustainable development. The local communities through their traditional leaders are managing some of their food requirements using indigenous knowledge technologies and know-how. These are proving to be useful and effective and compare favourably with alien technologies. Indigenous knowledge technologies such as the *Zunde raMambo* rely on locally available skills, materials, labour and are, thus, often more cost effective than exotic technologies introduced from the outside. Whilst there are many agricultural approaches that are peculiar to other environments and cultures and cannot easily be replicated elsewhere, the *Zunde* practice is consistent with many communities in Zimbabwe. *Zunde* agricultural practice has withstood the test of time and is not outdated regardless of the socio-economic and cultural changes that Zimbabweans have undergone since 1400 AD. The practice instils hard work amongst all members of society that include children and women. The *Zunde* practice does not hold that women and children should not work, but takes into consideration their knowledge during the implementation phase.

## Conclusion

Traditional communities rely on traditional knowledge. However, whilst indigenous knowledge is of great importance in the lives of the communities studied in all the four districts of Zimbabwe, little has been done to document it. It has been handed down from one generation to another through oral communication. It is evident that the *Zunde* practices in Hwedza, Guruve, Mangwe and Lupane have been employed successfully in addressing food insecurity. However, it is important to note that not all indigenous practices are beneficial to the DRR of a local community; and not all indigenous knowledge can *a priori* provide the right solution for a given problem. In this regard, any practice needs to be scrutinised for its appropriateness just as it occurs with any other technology.

A recommendation emanating from this study is that, when organising local people for projects to enhance agricultural resiliency to disasters mitigation, there is need to make effective use of traditional skills and knowledge that is embedded across the gender divide in that particular community. This may result in the improvement of prospects for community empowerment and self-reliance. The adoption of the bottom up participatory approach also encourages the highest levels of local participation in disaster risk reduction designed projects for the rural communities. The *Zunde* practice, for instance, provided valuable insight into the means with which communities and households interact. The Farmer Field Schools that were held under the *Zunde* practice, enabled farmers, village elders and chiefs to share ideas. These beneficiaries developed the skills and practices necessary to forge their own path for sustenance farming. Incorporating indigenous knowledge into disaster risk reduction policies can lead to the development of effective strategies that are cost effective, participatory and sustainable.

The study recommends the inclusion of this local knowledge into formal disaster risk reduction policy. On the basis of increased yields attributed to the technology of the *Zunde* practice, this should be cascaded to other areas that have similar cultural practices and climate. Embedded within this should be the promotion of the use of local resources and pooled labour to address the challenges of labour intensity associated with the *Zunde* practice. The Farmer Field School concept must be promoted as it produces expertise amongst the local farmers that is readily available and creates understanding about the local context. This eliminates the visiting experts concept in the community.
